# Mental imagery

**DOI:** 10.3389/fpsyg.2013.00198

**Published:** 2013-04-23

**Authors:** Joel Pearson, Stephen M. Kosslyn

**Affiliations:** ^1^School of Psychology, The University of New South WalesSydney, NSW, Australia; ^2^Minerva UniversitySan Francisco, CA, USA

Our ability to be conscious of the world around us is often discussed as one of the most amazing yet enigmatic processes under scientific investigation today. However, our ability to imagine the world around us in the absence of stimulation from that world is perhaps even more amazing.

Our capacity to re-experience objects or scenarios that we've encountered before, and to notice new things about those experiences, is itself remarkable. But perhaps more remarkable still is our ability to experience objects or events that do not exist in the world, through our imagination. This is perhaps one of the fundamental abilities that allow us successfully to plan, run dress rehearsals of future events, re-analyze the past—and even simulate or fantasize events that may never happen. In short, it could be argued that this ability is one of the main factors that have allowed us as a species to dominate our planet so profoundly.

**Figure:**
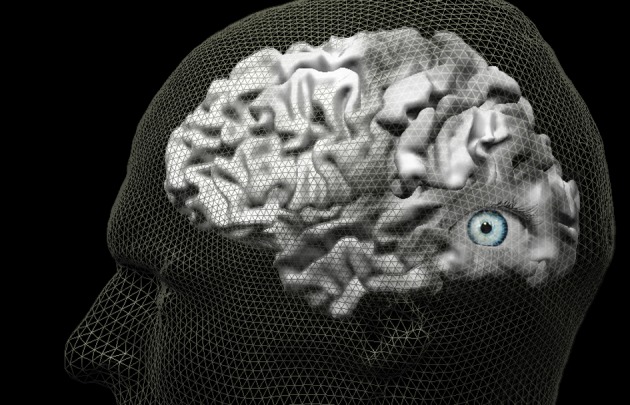


Empirical research into mental imagery has seen a recent surge, which is partly a result of new neuroscientific methods and their clever application—but is also due to the discovery and application of additional sorts of objective methods to investigate this inherently internal and private process.

Here we introduce an inspiringly broad range of work that focuses on mental imagery. This ebook contains the work from a broad range of researchers in different fields, both empirical work and reviews. Chapters range from the role of imagery in music, biomechanics, and mathematics to the functions of the cerebral hemispheres in imagery and imagery's effects on sensory perception.

This collection provides a cohesive and broad-spectrum addition to the rapidly growing field of mental imagery. This set of articles provides theoretical insights and an overview of the state of empirical understanding, where it is heading, and how mental imagery relates to other cognitive and sensory functions.

